# Supplementary material for: Transcriptome and Metabolome Analyses Reveal That Jasmonic Acids May Facilitate the Infection of Cucumber Green Mottle Mosaic Virus in Bottle Gourd
Source: Int J Mol Sci. 2023 Nov 21;24(23):16566. doi: 10.3390/ijms242316566 (PMC10706418; doi:10.3390/ijms242316566)
Supplement: Supplementary file 1 [file ijms-24-16566-s001.zip › Supplementary Figure S2.pdf]

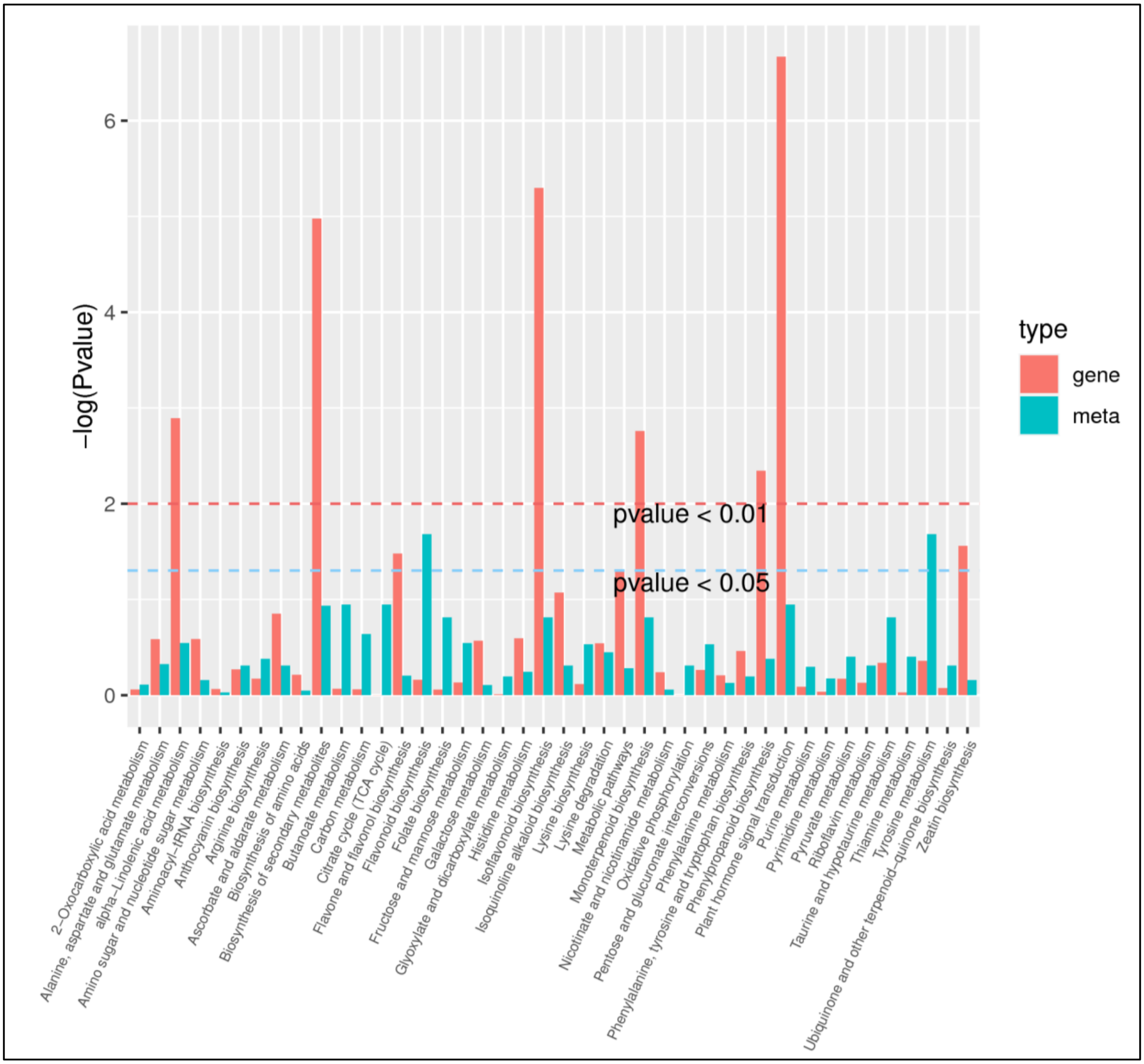

Supplementary Figure 2 KEGG enrichment analysis of transcriptome and metabolome.

The horizontal axis of the bar graph represents metabolic pathways, while the vertical axis in red represents the enrichment *p* value of differential genes and the green represents the enrichment *p* value of differential metabolites.
