# Supplementary material for: Transcriptome and Metabolome Analyses Reveal That Jasmonic Acids May Facilitate the Infection of Cucumber Green Mottle Mosaic Virus in Bottle Gourd
Source: Int J Mol Sci. 2023 Nov 21;24(23):16566. doi: 10.3390/ijms242316566 (PMC10706418; doi:10.3390/ijms242316566)
Supplement: Supplementary file 1 [file ijms-24-16566-s001.zip › Table S1.docx]

| **Table S1 \| Primers used in this study** | | |
| --- | --- | --- |
| **Primer** | **Primer sequence (5**'**-3**'**) ^a, b^** | **Purpose** |
| **CGMMV-CP-F** | ATGGCTTACAATCCGATCACACCT | RT-PCR detection of CGMMV |
| **CGMMV-CP-R** | CTAAGCTTTCGAGGTGGTAGCCT |  |
| **Lsi01G013470-F** | GCCAAGGACTACCAAGAC | qRT-PCR verification of RNA-seq result |
| **Lsi01G013470-R** | GCCAATAACAGTGAGAACAG |  |
| **Lsi02G017750-F** | GCTTCAGAATGTGTTGGTGAGATTGG | qRT-PCR verification of RNA-seq result |
| **Lsi02G017750-R** | ACGCTCCGATCATACGCCTACA |  |
| **Lsi05G011760-F** | AGAATACGAGGTCATAGAGCATCAAGTTC | qRT-PCR verification of RNA-seq result |
| **Lsi05G011760-R** | AGAATCACAGGATCTACGCCAATCATC |  |
| **Lsi04G015060-F** | GTCACTATTACTCAACCATCCAGATTGTC | qRT-PCR verification of RNA-seq result |
| **Lsi04G015060-R** | GATTAGGCTACGCAGGTAAGGAAGAT |  |
| **Lsi02G007470-F** | GCAACAAGGCTCTGGCAACTATCA | qRT-PCR verification of RNA-seq result |
| **Lsi02G007470-R** | GCTGGAGTCCTCTTCATAACTGTCATAC |  |
| **Lsi02G018990-F** | GCAACGGCGGAGGAAGTGAT | qRT-PCR verification of RNA-seq result |
| **Lsi02G018990-R** | ACATTCCCTTTACCATTCCCACCAAT |  |
| **Lsi01G009350-F** | CTCCAAATCCCTAACAAACTCAAATCCG | qRT-PCR verification of RNA-seq result |
| **Lsi01G009350-R** | GGCTCCAGTCACAATCACATCCAA |  |
| **Lsi02G021080-F** | TGGAAGAAGCAACACCATTATCACTG | qRT-PCR verification of RNA-seq result |
| **Lsi02G021080-R** | AGCAACGACTGTGGCGGAATT |  |
| **Lsi02G008430-F** | AGAGTTGATTGAAGATTGCTGGGATGAG | qRT-PCR verification of RNA-seq result |
| **Lsi02G008430-R** | CCTTCTATGGCTAAGACTGTGATGAATTGT |  |
| **Lsi11G012260-F** | AGACAGAGCATACATCCACCACCT | qRT-PCR verification of RNA-seq result |
| **Lsi11G012260-R** | ATCAACCTCCAGATCCTGAGCATTATTAG |  |
| **Lsi01G014560-F** | GCACCTGACATTCCGAGCACAT | qRT-PCR verification of RNA-seq result |
| **Lsi01G014560-R** | GAACACGAGCAACCTCCAGCAA |  |
| **Lsi04G002080-F** | CCAGTACCGCCATTGCCATCTC | qRT-PCR verification of RNA-seq result |
| **Lsi04G002080-R** | GGTGAAGATTCGCTAGGCTTGCT |  |
| **Lsi10G002560-F** | GTGCTTGTTGTTCTCGACGATGTG | qRT-PCR verification of RNA-seq result |
| **Lsi10G002560-R** | TCTCTTGTCGTTATGATGATCCTACTTCC |  |
| **Lsi11G005040-F** | TGGCATAGGAGGCATTGGTAAGACA | qRT-PCR verification of RNA-seq result |
| **Lsi11G005040-R** | TCCTGTAGTTGAACTAGACCATTGTATTGC |  |
| **Lsi05G018940-F** | TGTTACGACTTGTGAGGACGGATTC | qRT-PCR verification of RNA-seq result |
| **Lsi05G018940-R** | AGGACGCATGTTCAGAATACAGAGC |  |
| **Lsi05G012660-F** | CGCTTAGAGGTCTCACTGCTTGT | qRT-PCR verification of RNA-seq result |
| **Lsi05G012660-R** | CGCCGCTGCCTTCTGGATAT |  |
| **Lsi03G008210-F** | GGACATGCCGTCGTTCTTCTCTG | qRT-PCR verification of RNA-seq result |
| **Lsi03G008210-R** | CCTCTGTGTTGGACCACTGATTCAA |  |
| **Lsi09G006560-F** | TCTCGCAAACGCCCAATCTCTC | qRT-PCR verification of RNA-seq result |
| **Lsi09G006560-R** | CGCTGTGACAACGGAATTAACTCTC |  |
| **Lsi02G013460-F** | ATTCCTCCTCATCGCCGTCGTC | qRT-PCR verification of RNA-seq result |
| **Lsi02G013460-R** | ATCTTCTCCTTCGTCCATCAGCATCT |  |
| **Lsi11G000250-F** | GCAATGAGGAGCCTGGTTACACTT | qRT-PCR verification of RNA-seq result |
| **Lsi11G000250-R** | GATCTGACCGCCGAGCTTGTTC |  |
| **LOX-qRT-PCR-F** | GACCTAGCATCGCAAGGACCAAT | qRT-PCR |
| **LOX-qRT-PCR-R** | CTGTGCCACTGTAGAAGCCTGAG |  |
| **AOS-qRT-PCR-F** | CTACGATTACTTCTACATCCAAGGCAGAG | qRT-PCR |
| **AOS-qRT-PCR-F** | TGGCTCGGAAGACAGTGGAGTTAT |  |
| **AOC-qRT-PCR-F** | TGACCATTACGAGGCGATATACAGC | qRT-PCR |
| **AOC-qRT-PCR-R** | CCACCAGTCACAGCCAGATACG |  |
| **OPR3-qRT-PCR-F** | CCGACCAGTCACAAGCATTCCAAT | qRT-PCR |
| **OPR3-qRT-PCR-R** | CGACGGCAACGAGATTCAAGAGG |  |
| **JAR1-qRT-PCR-F** | CCGAAGTCAAGATTGGTGAAGAGTATG | qRT-PCR |
| **JAR1-qRT-PCR-R** | TCTGGCGTTGCGTTGTGGAA |  |
| **JMT-qRT-PCR-F** | GCGAATAACCGAGGAAGCAGTTGA | qRT-PCR |
| **JMT-qRT-PCR-R** | GCCCAGATGAGCAGCCCAAATC |  |
| **CYP94B3-qRT-PCR-F** | GACATGGCTCTTCTGGTTACTCACAA | qRT-PCR |
| **CYP94B3-qRT-PCR-R** | GAATCGTTGGACAATGAACTGGCTTC |  |
| **CYP94C1-qRT-PCR-F** | CTTGGAGCTGTCTCTTCCGATGTC | qRT-PCR |
| **CYP94C1-qRT-PCR-R** | TGTACCTAAGTTGAGCATTCGCTTGAT |  |
| **Myc2-qRT-PCR-F** | GTGGAAGCAGAACGACAGAGAAGG | qRT-PCR |
| **Myc2-qRT-PCR-R** | GCAACCGCATCAGATAGCAACGAT |  |
| **Myc3-qRT-PCR-F** | CCTCCGATTCCCTCTCTGATAATTCC | qRT-PCR |
| **Myc3-qRT-PCR-R** | CCGCTGTCGTTCTGCTTCCA |  |
| **Myb13-qRT-PCR-F** | CTAAGACAAGATTACGCCACCTCACA | qRT-PCR |
| **Myb13-qRT-PCR-R** | AGATTCTTCTTCACCGCCGCAAAT |  |
| **Myb48-qRT-PCR-F** | TCTGTGGAGAATGGAGGAGGAAGAA | qRT-PCR |
| **Myb48-qRT-PCR-R** | TCTGATTAGGCTGAGCTAAACCAAGG |  |
| **Myb62-qRT-PCR-F** | AGAAGACAATCTCCTCATCCATTCCA | qRT-PCR |
| **Myb62-qRT-PCR-R** | AATTAAGCCACCTCAATCTGCAACTC |  |
| **Myb77-qRT-PCR-F** | GACGATCCATTGACGGCACTAACC | qRT-PCR |
| **Myb77-qRT-PCR-R** | TACTCTCTCACTTCCCTCGCTACAAC |  |
| **Myb86-qRT-PCR-F** | AGCAGCTCAATTACCAGGAAGAACAG | qRT-PCR |
| **Myb86-qRT-PCR-R** | CTCCTCCAATGGCTTGTGAGTTGT |  |
| **NAC-qRT-PCR-F** | GACGACCGAGTTGACTCAGATGC | qRT-PCR |
| **NAC-qRT-PCR-R** | CGGAACGGCGATTGGATGAGAC |  |
| **WRKY6-qRT-PCR-F** | GGTTCAGTCGTCGGAGCAATCG | qRT-PCR |
| **WRKY6-qRT-PCR-R** | GTTATCAAGCGAGAAATCCCAATCCC |  |
| **WRKY18-qRT-PCR-F** | GCTATTCCTCCTGGCTTGAGTCCT | qRT-PCR |
| **WRKY18-qRT-PCR-R** | GGCGACGGCAGAACATGAGAAG |  |
| **WRKY31-qRT-PCR-F** | GCCGATCACGACACCACTACCA | qRT-PCR |
| **WRKY31-qRT-PCR-F** | GCCACCGACGAGTTGATTGTTAGAA |  |
| **WRKY68-qRT-PCR-F** | GAACTCACAACCATCCTCTACCTGTT | qRT-PCR |
| **WRKY68-qRT-PCR-R** | CGGCAGTAGCGGCGAATGAA |  |
| **ERF1-qRT-PCR-F** | CACCGCCGCCATTCTCAACT | qRT-PCR |
| **ERF1-qRT-PCR-R** | GAGGAAGAGGAGGAGGAAGAGGAA |  |
| **ERF1b-qRT-PCR-F** | TTAATCTCCGAGGCTGCCGTCTA | qRT-PCR |
| **ERF1b-qRT-PCR-R** | GGGTCTTCTTCTCCGAGCGAATC |  |
| **ERFAP2-qRT-PCR-F** | GAATAGAGGTGGAATTGTGGACTTCAGAG | qRT-PCR |
| **ERFAP2-qRT-PCR-R** | ACGGCGGAGATGGTGTTACTTGA |  |
| **ERF2-qRT-PCR-F** | TTGGCTCACGGCTCGGCTAA | qRT-PCR |
| **ERF2-qRT-PCR-R** | CAACTGGCAACACTGACATCTGG |  |
| **ERF2b-qRT-PCR-F** | AGAGGAAGAGAACGAGGTGGAGAA | qRT-PCR |
| **ERF2b-qRT-PCR-R** | CGAGGTACGGAATCTGGTAGAACTT |  |
| **ERF6-qRT-PCR-F** | TCAGGCGGAGAAGATCAGTGACTT | qRT-PCR |
| **ERF6-qRT-PCR-R** | TCAACTACGGATGACGAATCGGAGT |  |
