# Supplementary figures and images for: Transcriptome and Metabolome Analyses Reveal That Jasmonic Acids May Facilitate the Infection of Cucumber Green Mottle Mosaic Virus in Bottle Gourd
Source: Int J Mol Sci. 2023 Nov 21;24(23):16566. doi: 10.3390/ijms242316566 (PMC10706418; doi:10.3390/ijms242316566)

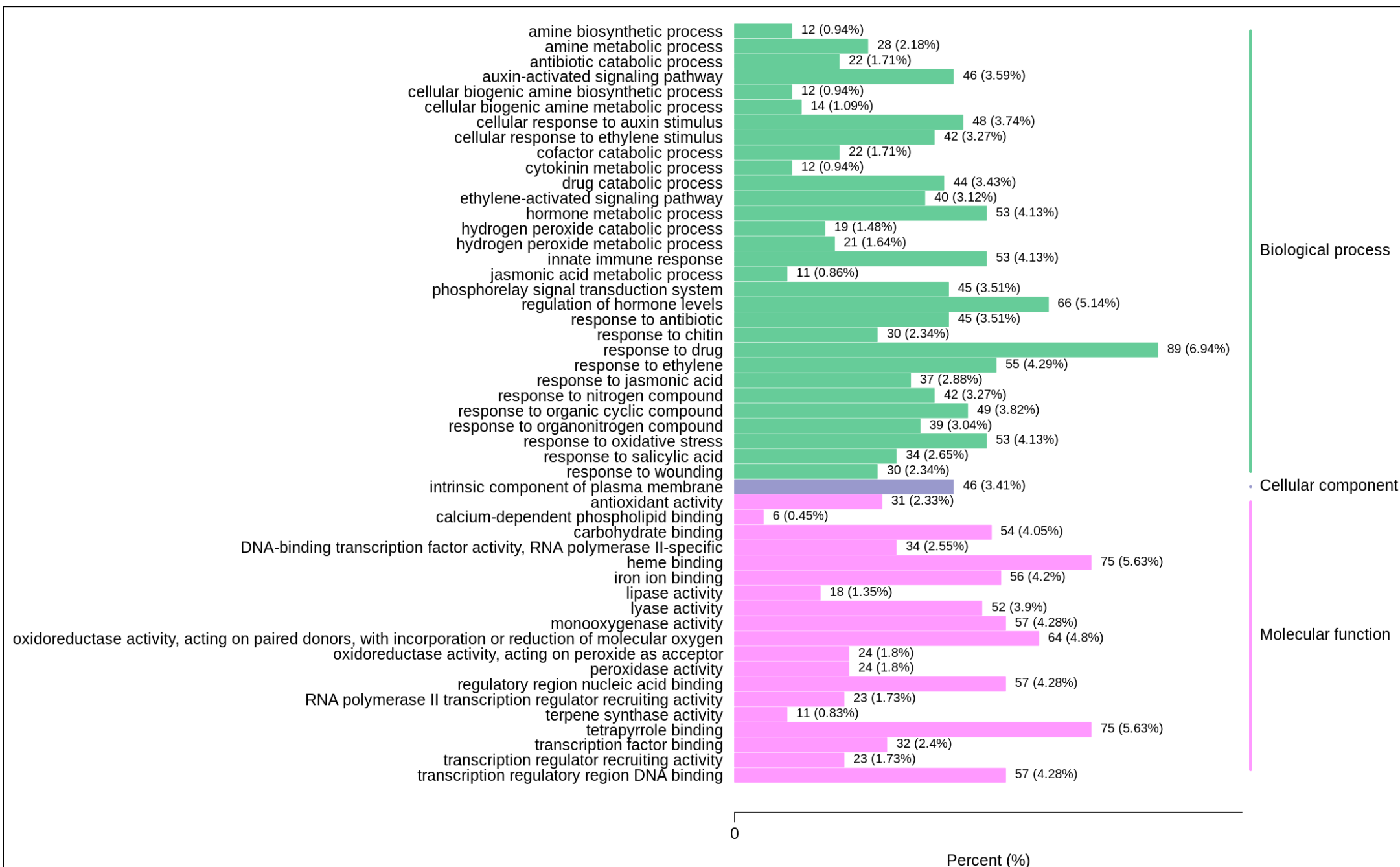

**Supplementary Figure 1 Go enrichment analysis of DEGs between ZLM12 and ZLV12**

Supplement: Supplementary file 1 [file ijms-24-16566-s001.zip › Supplementary Figure S1.pdf]
